# Minimal gene set discovery in single-cell mRNA-seq datasets with ActiveSVM

**DOI:** 10.1038/s43588-022-00263-8

**Published:** 2022-06-27

**Authors:** Xiaoqiao Chen, Sisi Chen, Matt Thomson

**Affiliations:** 1 Department of Computing and Mathematical Sciences, California Institute of Technology, Pasadena, California USA; 2 Division of Biology and Biological Engineering, California Institute of Technology, Pasadena, California USA; 3 Beckman Institute Single-cell Profiling and Engineering Center, Pasadena, California USA

**Keywords:** Machine learning, Computational models, RNA sequencing, Gene expression

## Abstract

Sequencing costs currently prohibit the application of single-cell mRNA-seq to many biological and clinical analyses. Targeted single-cell mRNA-sequencing reduces sequencing costs by profiling reduced gene sets that capture biological information with a minimal number of genes. Here we introduce an active learning method that identifies minimal but highly informative gene sets that enable the identification of cell types, physiological states and genetic perturbations in single-cell data using a small number of genes. Our active feature selection procedure generates minimal gene sets from single-cell data by employing an active support vector machine (ActiveSVM) classifier. We demonstrate that ActiveSVM feature selection identifies gene sets that enable ~90% cell-type classification accuracy across, for example, cell atlas and disease-characterization datasets. The discovery of small but highly informative gene sets should enable reductions in the number of measurements necessary for application of single-cell mRNA-seq to clinical tests, therapeutic discovery and genetic screens.

## Abstract (web summary)

ActiveSVM is introduced as a feature selection procedure for discovering minimal gene sets in large single-cell mRNA-seq datasets.

## Main

Single-cell mRNA-seq methods have scaled to allow routine transcriptome-scale profiling of thousands of cells per experimental run. Although single cell mRNA-seq approaches provide insights into many different biological and biomedical problems, high sequencing costs prohibit the broad application of single-cell mRNA-seq in many exploratory assays such as small-molecule and genetic screens, and in cost-sensitive clinical assays. The sequencing bottleneck has led to the development of targeted mRNA-seq strategies that reduce sequencing costs by up to 90% by focusing sequencing resources on highly informative genes for a given biological question or an analysis^1–5^. Commercial gene-targeting kits, for example, reduce sequencing costs through selective amplification of specific transcripts using ~1,000 gene-targeting primers.

Cells modulate gene expression through the regulation of transcriptional programs or modules that contain multiple genes regulated by common sets of transcription factors^1^. Genes within transcriptional modules exhibit correlated gene expression due to co-regulation. Correlations in gene expression can enable the transcriptional state of a cell to be reconstructed through the targeted mRNA profiling of a small number of highly informative genes^1,3^. However, such targeted sequencing approaches require computational methods to identify highly informative genes for specific biological questions, systems or conditions. A range of computational approaches, including differential gene expression analysis and principal components analysis (PCA), can be applied to identify highly informative genes^1^. Yet, current methods for defining minimal gene sets are computationally expensive to apply to large single-cell mRNA-seq datasets and often require heuristic user-defined thresholds for gene selection^6,7^. As an example, computational approaches based on matrix factorization (PCA, non-negative matrix factorization) are typically applied to complete datasets and therefore are computationally intensive when datasets scale into the millions of cells^8^. Furthermore, gene set selection after matrix factorization requires heuristic strategies for thresholding coefficients in gene vectors extracted by PCA or non-negative matrix factorization, and then querying whether the selected genes retain core biological information.

Inspired by active learning^9^ approaches, here we develop a computational method that selects minimal gene sets capable of reliably identifying cell types and transcriptional states through an active support vector machine classification task (ActiveSVM)^10,11^. The ActiveSVM algorithm constructs a minimal gene set through an iterative cell-state classification task. At each iteration, ActiveSVM applies the current gene set to classify cells into classes that are provided by unsupervised clustering of cell states, or by supplied experimental labels. The procedure analyzes cells that are misclassified with the current gene set and then identifies maximally informative genes that are added to the growing gene set to improve classification. Traditional active learning algorithms query an oracle for training examples that meet a criteria^12^. The ActiveSVM procedure actively queries the output of an SVM classifier for cells that classify poorly, and then performs a detailed analysis of the misclassified cells to select maximally informative genes. By selecting minimal gene sets through a well-defined classification task, we ensure that the gene sets discovered by ActiveSVM retain biological information.

The central contribution of ActiveSVM is that the method can scale to large single-cell datasets with more than one million cells as the procedure focuses computational resources on poorly classified cells. As the algorithm only analyzes the full transcriptome of cells that classify poorly with the current gene set, the method can be applied to discover small sets of genes that can distinguish between cell types at high accuracy even in datasets with over a million profiled cells. We demonstrate that ActiveSVM can analyze a mouse brain dataset with 1.3 million cells in only hours of computational time. In addition to scaling, the ActiveSVM classification paradigm generalizes to a range of single-cell data analysis tasks, including the identification of disease markers, genes that respond to Cas9 perturbation and region-specific genes in spatial transcriptomics.

To demonstrate the performance of ActiveSVM, we apply the method to a series of single-cell genomics datasets and analysis tasks. We identify minimal gene sets for cell-state classification in human peripheral blood mononuclear cells (PBMCs)^13^, the megacell mouse brain dataset^14^, and the Tabula Muris mouse tissue survey^15^. We identify disease markers that distinguish healthy and multiple myeloma patient PBMCs^16^. To highlight the generality of the method, we apply ActiveSVM to identify genes impacted by Cas9-based gene-knock down in perturb-seq^17^ and demonstrate that ActiveSVM can identify gene sets that mark specific spatial locations of a tissue through analysis of spatial transcriptomics data^18^. Gene sets constructed by ActiveSVM are both small and highly efficient, for example, classifying human immune cell types within PMBCs using as few as 15 genes and classifying 55 cell-states in Tabula Muris with <150 genes. The gene sets we discover include both classical markers and genes not previously established as canonical cell-state markers. Conceptually, ActiveSVM demonstrates that active sampling strategies can be applied to enable the scaling of algorithms to the large datasets generated single-cell genomics.

## Results

### Overview of ActiveSVM feature selection

**Fig. 1:**
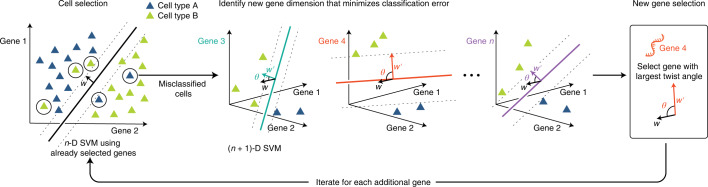
Description of ActiveSVM feature selection. At the *n*th step, an *n*-D SVM using only already-selected genes is trained to select a certain number of misclassified cells, which is the cell selection step. In the gene selection step, the least classifiable cells are taken as the training set. Based on this training set, *N* – *n* (*n* + 1)-D SVMs are trained, where *n* dimensions are the genes already selected and the last dimension is one of the previously unselected candidate genes. We would then obtain *N* – *n* weights $w^{\prime}$ corresponding to *N* – *n* unselected genes as well as *N* – *n* margin rotation angles *θ* between every $w^{\prime}$ and the original weight *w* of the *n*-D SVM. The gene with the maximum rotation of margin is selected for the next round.

We developed a computational that applies a support vector machine classifier to identify compact gene sets that distinguish cell-states in single-cell data (Fig. 1 and Supplementary Algorithm 1). The ActiveSVM procedure starts with an empty gene set, an empty cell set, and a list of candidate genes and cells. The algorithm iteratively selects genes and classifies cells using identified genes by training a SVM model to classify the cell types according to labels. The algorithm identifies cells in the dataset that classify poorly given the current gene set, and uses misclassified cells to select additional genes to improve classification accuracy on the entire dataset. Gene selection specifically identifies genes that will maximally rotate the SVM margin (see Methods).

ActiveSVM constructs minimal gene sets through iterative rounds of classification and gene selection based on a set of cell labels. The computational efficiency of the algorithm emerges because ActiveSVM only performs full transcriptome analysis on cells that classify poorly given the current gene set. The cell-classification and gene selection strategies are discussed formally in the Methods. ActiveSVM depends on cell labels that can be derived from unsupervised analysis, experimental metadata or biological knowledge of cell-type marker genes. We supply min-complexity and min-cell versions of ActiveSVM algorithm. The min-complexity algorithm samples a fixed number of misclassified cells and directly uses them as the cell set to select the next gene. The min-cell algorithm reuses the misclassified cells selected in previous iterations to reduce the total number of required cells.

### Identifying minimal gene sets in single-cell mRNA-seq data with ActiveSVM

We tested our ActiveSVM feature selection method on four single-cell mRNA-seq datasets: a dataset of PBMCs^13^, the megacell 1.3-million-cell mouse brain dataset^14^, the Tabula Muris mouse tissue survey dataset^15^ and a multiple myeloma human disease dataset^16^. We later demonstrated generalization of the strategy to additional types of single-cell data analysis, including a perturb-seq dataset where genes impacted by Cas9-based genetic perturbation^17^ and a spatial transcriptomics dataset by seqFish+ (ref. ^18^).

For each analysis, we show the classification accuracy of the test set along with the number of genes we select. We also compare the classification performance to several widely used feature selection methods, including conventional SVM and strategies that apply correlation coefficients, mutual information^19^, a $\chi^2$ test^20^, feature importance by decision tree^21^ and uniform random sampling of genes (the null strategy), showing that ActiveSVM obtains the highest accuracy. Additionally, ActiveSVM considerably reduces time and memory consumption, especially for large datasets such as the megacell 1.3-million-cell mouse brain dataset (Supplementary Table 1). All of the comparison methods select genes one by one and select a new gene with the largest score in terms of the corresponding evaluation functions while using the same number of cells as our method. However, all methods randomly sample cells at each iteration without an active learning approach. In each experiment, the dataset was first preprocessed and normalized using standard single-cell genomics strategies (see Methods). The details about algorithm parameters optimization are provided in the Supplementary Information, and the parameters for each dataset are given in Supplementary Tables 2–4. The entire dataset was then randomly split into training and test sets with the sizes of 80% and 20%, respectively.

### Active feature selection on human PBMC data

To test the performance of ActiveSVM, we applied the method to extract classifying gene subsets for human PBMCs. We analyzed a single-cell transcriptional profiling dataset for 10,194 cells^13^ with 6,915 genes. We used Louvain clustering^22^ to identify T-cells, activated T and NK cells, B-cells and monocytes.

**Fig. 2:**
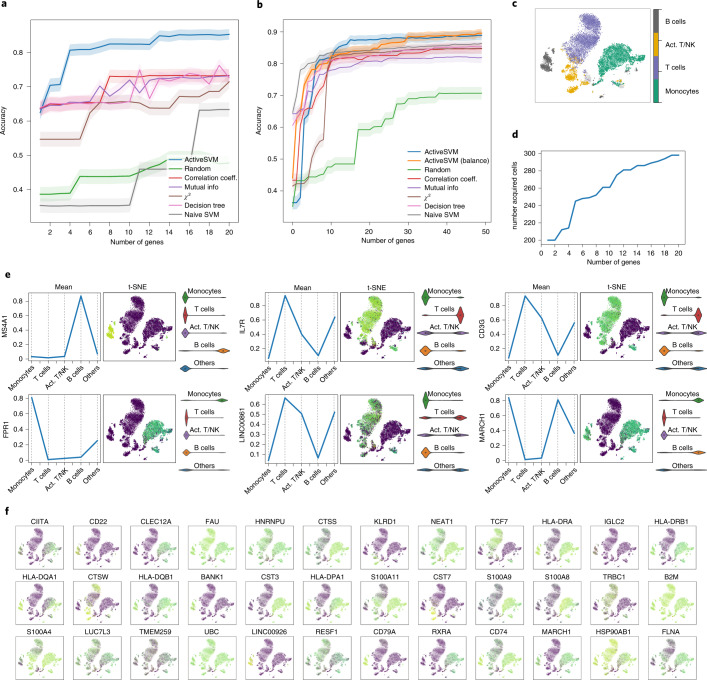
Gene selection and cell-type classification for PBMC dataset. **a**, The test accuracy for min-cell strategy and a series of comparison classification strategies. The min-cell strategy selects *k* = 20 genes and select *c* = 100 cells each iteration with confidence interval estimates. **b**, The test accuracy of min-complexity strategy that selects *k* = 50 genes using *c* = 20 cells each iteration. **c**, The t-SNE plots of the entire filtered dataset. In the legend, Act. T/NK refers to activated T and NK cells. **d**, The total number of unique cells used versus gene set size with the min-cell strategy. **e**, Plots showing the expression of several genes markers, including mean on classes, gene expression value on t-SNE projection, and violin plots. **f**, Expression level of additional selected genes overlaid on t-SNE plot. Source data

Both the min-cell and min complexity strategies identified gene sets that classify the five major cell-types at greater than 85% accuracy with as few as 15 total genes (Fig. 2a–c). In addition to enabling cell-type classification of the dataset, the ActiveSVM gene sets provide a low-dimensional space in which to analyze the data. A key benefit of the active learning strategy is that a relatively small fraction of the dataset is analyzed, so that the procedure can generate the gene sets while only analyzing 298 cells (Fig. 2d). In the min-cell strategy, at each iteration, a specific number of misclassified cells (*c* = 100) are selected but the total number of cells used does not increase in increments of 100, as some cells are repeatedly misclassified and are thus repeatedly used for each iteration.

The ActiveSVM procedure generates gene sets that contain known markers, each plotted individually in a t-SNE grid (Fig. 2e,f). For instance, MS4A1 and CD79 are well-established B-cell markers, and IL7R and CD3G are well-established T-cell markers. However, we also find genes that are not commonly used as markers, but whose expression is cell-type specific. For instance, we find highly monocyte-specific expression of FPR1, which encodes an *N*-formyl peptide receptor recently discovered to be the receptor for plague effector proteins^23^. We also find T-cell/NK-cell specific expression of a long non-coding RNA, LINC00861, whose function is unknown but has been correlated with better patient outcome in lung adenocarcinoma^24^. The marker genes are generally highly specific for individual cell types, but some mark multiple cell types (for example, MARCH1, which marks monocytes and B-cells).

### Scaling of ActiveSVM to million cell, mouse brain dataset

To demonstrate the scaling of the ActiveSVM feature selection method to large single-cell mRNA-seq datasets, we applied the method to extract compact gene sets from the the megacell demonstration dataset^14^, which was collected by 10x Genomics as a scaling demonstration of their droplet scRNA-seq technology. The dataset contains full transcriptome mRNA-seq data for 1.3 million cells from the developing mouse brain profiled at embryonic day 18^14^. The dataset is one of the largest single cell mRNA-seq datasets currently available. The size of the dataset has been a challenge for data analysis, and a previous analysis paper was published that developed subsampling methods that extract marker genes and cell-types by extracting subsets of of a dataset containing ~100,000 cells^8^. We found that it was possible to run ActiveSVM on a conventional laptop. We analyzed the megacell dataset on an AWS instance r5n.24xlarge to decrease computational time, on which ActiveSVM ran in 69 min and 243 min for the min-complexity and min-cell strategies, respectively. By comparison, the other methods listed required more than four days to run all 1.3 million cells on the same AWS instance (Supplementary Table 1); furthermore, ActiveSVM's peak memory usage is 2,111 MB, whereas the other methods all consume more than 78,600 MB in the same AWS instance (Supplementary Table 1).

**Fig. 3:**
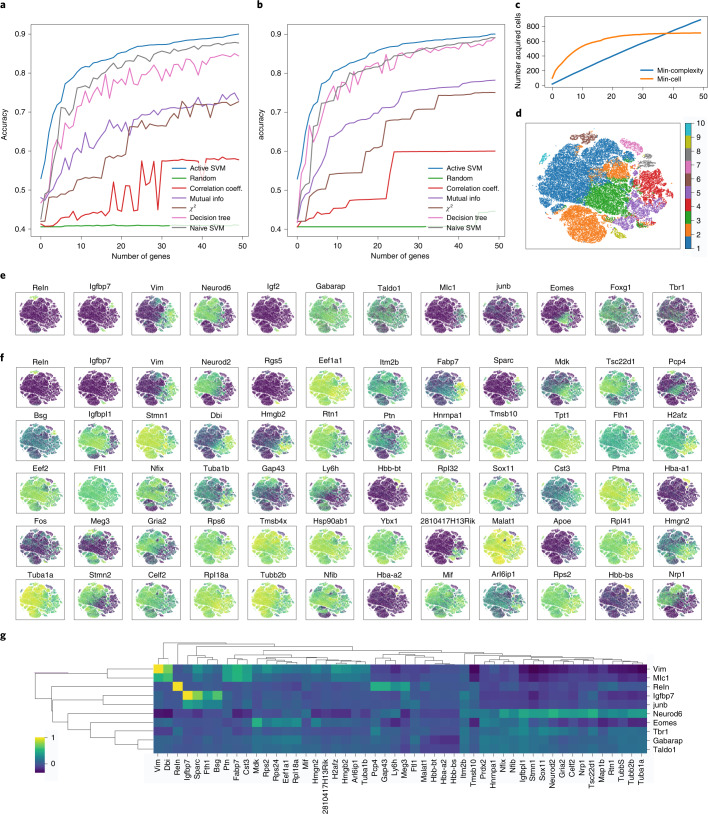
Scaling of ActiveSVM feature selection to 1.3-million-cell mouse brain dataset. **a**, The test accuracy of the min-complexity strategy that selects 50 genes using 20 cells each iteration. **b**, The test accuracy of the min-cell strategy that selects 50 genes using 100 cells each iteration. **c**, The total number of unique cells used versus gene set size with both the min-complexity and min-cell strategies. **d**, The t-SNE plots of the entire filtered dataset with ten classes by *k*-means clustering. **e**, Expression level of the gene markers from previously published analysis overlaid on a t-SNE plot. **f**, Expression level of the gene markers selected by ActiveSVM overlaid on a t-SNE plot, where the first row are the genes that have similar distribution with gene markers from previously analysis and other genes are new markers correlated with the classification target. **g**, Correlation matrix of literature markers (*y*-axis) from ref. ^8^ versus ActiveSVM selected genes (*x*-axis). Source data

On the megacell dataset, the ActiveSVM procedure discovered gene sets that achieve ~90% classification accuracy with only 50 genes while analyzing fewer than 1,000 cells (Fig. 3a–c). The procedure discovered a series of cell-state specific marker genes that extend prior analysis (Fig. 3d–f). For example, the analysis in ref. ^8^ identified marker genes through subsampling and a past biological work, and the ActiveSVM analysis discovered several of the same markers as the past work (Reln, Vim, Igfbp7) (Fig. 3e,f). Furthermore, ActiveSVM extended on the work by identifying additional markers that correlate with the previous analysis, as well as the marker genes of additional cell states (Fig. 3g). The development of radial glial cells in particular has been of intense recent interest as they are the stem cells of the mouse and human neocortices^25^. Careful molecular analysis has defined markers of radial glial cells such as Vim. ActiveSVM identified a group of genes whose expression correlates with Vim across the mouse brain at embryonic day 18, including diazepam binding inhibitor (Dbi, an Acyl-CoA binding protein), Hmgb2 and Ptn^8^. The Vim-correlated gene network includes additional transcription factors, Hmgb2^25^, as well as a core group of genes, Ptn and Fabp7 (which are also brain lipid binding proteins), and two components of a radial glia-signaling network^25–27^, which has been identified as a core regulatory module supporting the proliferation and stem cell state in the radial glial cell population.

The neural progenitor transcription factor Neurod6 marked a separate cell population that we identified to contain genes such as Neurod2 and Sox11 (transcription factors), Nfib and Nfix (glial transcription factors), and the receptor glutamate ionotropic receptor AMPA-type subunit 2 (Gria2) (Fig. 3e–g). The marker genes observed in Neurod6-expressing cells were anticorrelated with the Vim-correlated markers, which suggests that ActiveSVM identified two distinct regulatory modules. Structurally, the tubulin proteins Tuba1b and Tuba1a were expressed in Vim and Neurod6 populations, respectively. In addition to genes correlated or anticorrelated with existing markers, ActiveSVM identified markers of additional cell populations, such as Meg3, a long non-coding RNA expressed in cluster 2.

Broadly, the analysis of the megacell mouse brain dataset demonstrates that ActiveSVM scales to analyze a large dataset with >1 million cells. The analysis of such large datasets has been challenging with conventional approaches that attempt to store the entire set in memory for analysis. Past analysis of the 10x Genomics Megacell dataset found that subsamples with greater than 100,000 cells would yield an out-of-memory error on a server node with 64 cores, a 2.6 GHz processor and 512 GB of RAM^8^. Through iterative analysis, ActiveSVM identifies known marker and regulatory genes, genes that correlate with known markers, and genes of additional cell populations that could provide a starting point for future experimental investigations.

### Gene sets for cell-type classification in mouse tissue survey

In addition to analyzing a dataset with a large number of total cells, we sought to benchmark ActiveSVM's feature selection performance on a dataset with a large number of distinct cell types. We applied ActiveSVM to the Tabula Muris mouse tissue survey, a droplet-based scRNA-sequencing dataset that contains 55,656 single cells across 58 annotated cell types and 12 major tissues^15^. For each cell, 8,661 genes are measured. We used the supplied cell-type labels in our analysis, agnostic of tissue type. Cells labeled macrophage from the spleen are thus considered to belong to the same class as those labeled macrophage from the mammary gland.

**Fig. 4:**
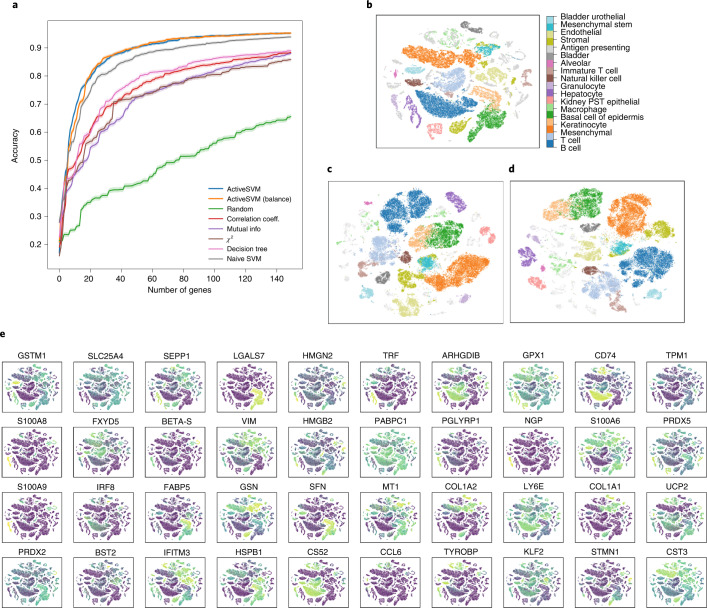
Minimal gene sets for cell-type classification in the Tabula Muris mouse tissue survey. Classification results of 150 genes selected using the min-complexity strategy with 20 cells each iteration. **a**–**e**, The subplots contain: classification accuracy versus gene set size (**a**); the t-SNE plots of the entire filtered dataset (**b**); the t-SNE plots of the gene set selected with randomly sampling (**c**); the t-SNE plots of the gene set selected with balanced sampling (**d**); and the expression level overlaid on t-SNE projection for genes selected (**e**). Source data

Even with a large number of cell types, ActiveSVM can construct gene sets that achieve high accuracy (>90%) compared with other methods (Fig. 4a and Supplementary Fig. 1a). To construct a gene set of size 500, ActiveSVM feature selection used fewer than 800 unique cells (Supplementary Fig. 1c) or an average of 14 cells per cell type. We were able to recreate the clustering patterns from the original data (Fig. 4b and Supplementary Fig. 1b) when analyzing the cells within the low-dimensional t-SNE space spanned by the selected 150 genes (Fig. 4c,d) or 500 genes (Supplementary Fig. 1d).

Our approach allowed us to construct a set of marker genes able to identify mouse cell types across disparate tissues (Fig. 4e and Supplementary Fig. 1e). Even when analyzing a large number of cell types, we were able to identify highly cell type-specific genes such as: (1) CD3D, a well-established T-cell marker; (2) transferrin (TRF), which is selectively secreted by hepatocytes^28^ or (3) galectin-7 (LGALS7), which is specific for basal and differentiated cells of stratified epithelium^29^. However, given the functional overlap between different cell types, the genes within our set include many that mark multiple cell types. For instance, H2-EB1^30^, a protein important in antigen presentation, is expressed in B-cells and macrophages, both of which are professional antigen presenting cells. Our analysis also identified cell type-specific expression for a number of poorly studied genes, such as granulocyte- and hepatocyte-specific expression of 1100001G20RIK (also known as Wdnm-like adipokine), which has previously only been associated with adipocytes^31^.

### Minimal gene sets for identifying multiple myeloma patients

To analyze ActiveSVM as a tool for the discovery of disease-specific markers, we used single-cell data from peripheral blood immune cells collected from two healthy donors and four patients who have been diagnosed with multiple myeloma^16^, an incurable cancer of plasma cells (known as myeloma cells) that over-proliferate in the bone marrow. Although myeloma cells are typically the target of analysis as they are the causative agent of disease, peripherally circulating immune cells also contain signatures of disease, including a depleted B-cell population^32,33^, an increased myeloid-derived suppressor cell count^34^ and T-cell immunosenescence^33,35^. We sought to further define transcriptional markers that distinguish healthy peripheral immune cells from the cells of multiple myeloma patients. We performed feature selection using heterogeneous populations of cells labeled only by disease state; the dataset contains 35,159 cells with 32,527 genes.

**Fig. 5:**
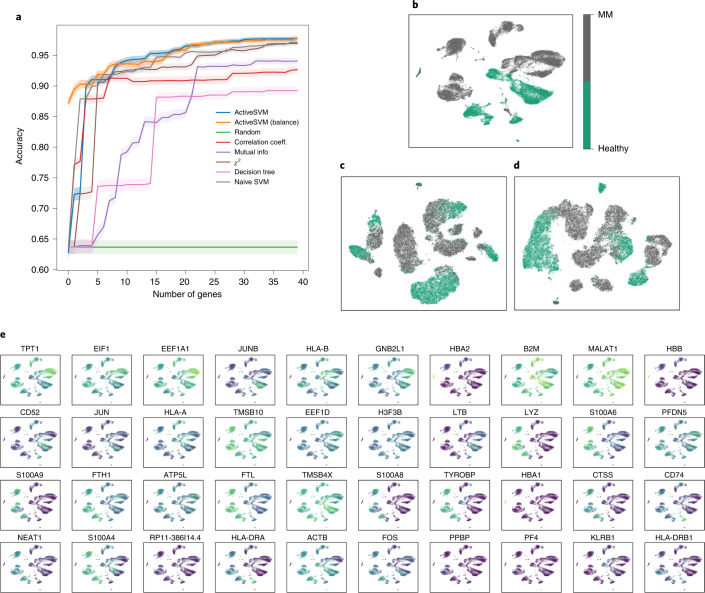
Gene set selection for healthy versus disease classification in multiple myeloma dataset. Classification results of 40 genes selected by min-complexity strategy using 20 cells each iteration. **a**, Classification accuracy versus gene set size. **b**, The t-SNE plots of the entire filtered dataset. **c**, The t-SNE plots of the gene set selected with randomly sampling. **d**, The t-SNE plots of the gene set selected with `balanced' sampling. **e**, The expression level overlaid on t-SNE projection for genes selected. Source data

We compared the classification accuracy for ActiveSVM with the other methods (Fig. 5a and Supplementary Fig. 2a) and found that ActiveSVM achieved high accuracy in a limited number of steps and consistently outperformed the other methods using both random and balanced sampling. Non-overlapping cell-type clusters were identified for healthy and multiple myeloma cells in the original dataset in t-SNE projections (Fig. 5b and Supplementary Fig. 2b). As few as 449 cells are acquired when using the min-cell strategy (Supplementary Fig. 2c). The non-overlapping clusters are replicated in t-SNEs constructed from 40 genes selected using both the min-complexity (Fig. 5c,d) and min-cell (Supplementary Fig. 2d) strategies. Minimal gene sets were sufficient to separate multiple myeloma from healthy samples in t-SNE representations of the data using the min-complexity strategy with and without cell balancing.

ActiveSVM identified both known and markers of multiple myeloma within the peripheral blood immune cells (Fig. 5e and Supplementary Fig. 2e). Our analysis identified TPT1, which has been associated with multiple myeloma in the past^36^, and RACK1 (also known as GNB2L1), a scaffolding protein that coordinates critical functions such as cell motility, survival and death, and is broadly upregulated in peripheral immune cells from multiple myeloma patients. Although this gene has been previously associated with myeloma cells^37^, its regulation had not been reported in peripherally circulating immune cells. The procedure also identifies multiple members of the S100 calcium-binding protein family (S100A8, S100A9, S100A6 and S10084)^38–40^ as members of the genes sets that separate multiple myeloma versus healthy samples. The S100 protein family defines a module of genes that are associated with the induction of stress-response pathways. A recent study found that S100A4 expression correlates with poor patient survival in mulitple myeloma, and that S100A8 and S100A9 are markers that correlate with poor response of multiple myeloma patients to treatment with proteasome inhibitors and the histone deacetylase inhibitor panobinostat^39^. The result demonstrates that ActiveSVM can automatically define groups of genes that have clinical association with disease progression and treatment outcome. The minimal gene sets generated by ActiveSVM could provide useful targeted sequencing panels for a variety of clinical tasks.

### Identifying genes impacted by Cas9 perturbation

The analyses above demonstrated that ActiveSVM identifies minimal gene sets for cell-state identification across a range of single-cell mRNA-seq datasets. We next demonstrate that ActiveSVM provides a more general analysis tool, with potential applications to a range of single-cell genomics analysis tasks. To demonstrate generalization of ActiveSVM-based gene set selection across single-cell genomics tasks, we applied the method to identify marker genes in two additional applications: perturb-seq and spatial transcriptomics.

Perturb-seq is an experimental method for performing Cas9-based genetic screens with single-cell mRNA-seq read-outs. In perturb-seq, cells are induced with pooled libraries of guide RNA’s that target the Cas9 protein to cut and silence specific genes^3,17^. Individual cells stochastically take-up specific guide RNAs, whereas Cas9 cuts and silences targeted genes in the genome. Following the perturbation experiment, single-cell mRNA-seq is applied to read both the transcriptome of each cell and the identity of the delivered sgRNA through sequencing. The advantage of the perturb-seq method is that many knock-out experiments can be performed simultaneously. However, a challenge is that noise impacts the measurement of guide RNA identify and, furthermore, the cutting of the genome by the Cas9 molecule is not complete. Due to measurement and experimental noise, identifying the impact of genetic perturbation on a cell population can be challenging, and various methods have been developed to boost signal^3^.

**Fig. 6:**
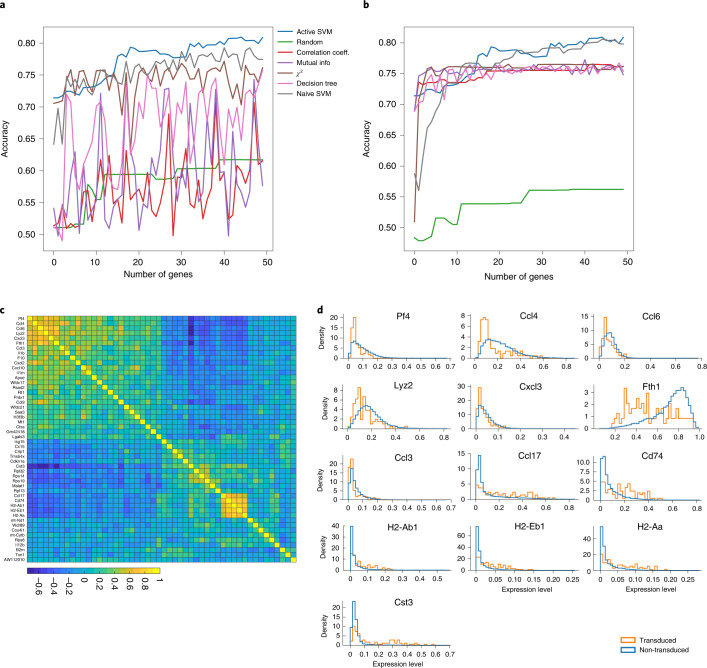
Application of ActiveSVM to identify genes expression changes following Cebp knock-down with perturb-seq. The results of classification on perturb-seq data where cells are labeled and classified as Cebp sgRNA transduced or not transduced with a guide RNA. **a**,**b**, Accuracy of entire dataset with min-complexity strategy, where comparison methods use the same number of cells as ActiveSVM (**a**), and use the entire dataset (**b**). **c**, Correlation matrix showing pair-wise correlation coefficients for genes in Cebp-perturbed cells. The correlation matrix identifies two gene modules. **d**, Distributions of gene expression in Cebp sgRNA transduced (orange) or not transduced (blue) cells. Selected genes from modules in **c** shown and organized so that genes whose expression increases with Cebp perturbation are on top and repressed genes are on the bottom of the figure. Source data

We applied ActiveSVM to identify a minimal gene set as well as downstream effects of transcription factor knock-down in perturb-seq data collected from mouse dendritic cells with transcription factor knock-downs^17^. We focused our analysis on knock-down Cebp (a pioneering transcription factor in mouse dendritic cells) stimulated for 3 h with lipopolysaccharide (LPS), a signal that mimics bacterial infection. ActiveSVM identified minimal gene sets (50 genes) that achieved about 80% classification accuracy on the Cebp sgRNA cell label with the class-balancing strategy. ActiveSVM performed better than the competing methods on this noisy dataset, even if ActiveSVM only used a small subset of data while comparison methods performed on the entire dataset (Fig. 6a,b).

The discovered gene set contains two modules of correlated genes (Fig. 6c,d). Gene expression distributions for cells in transduced versus non-transduced cells demonstrated that the modules represented two groups of genes. One group (including Pf4, Ccl4, Ccl6, Lyz2) was repressed by Cebp knock-down, whereas the other (including Ccl17, Cd74, H2-Ab1) was activated by Cebp knock-down (Fig. 6d). In both cases, the identified gene sets contained known targets of Cebp, the perturbed transcription factor. For example, ferritin, heavy polypeptide 1 (Fth1), Cst3, Tmsb4x, Lgals3, Ccl4 and Cd74 are all previously identified as direct binding targets of Cebp as determined by Chip-seq^41^. As Cebp knock-down leads to both up- and down-regulation of genes, the results suggest that the factor can play both activating and repressive roles consistent with prior literature^42^.

Our analysis of the Perturb-seq data demonstrates that ActiveSVM can be applied as a useful tool for the identification of genes modulated by perturb-seq experiments. ActiveSVM can return minimal genes sets that contain functional information. Moreover, perturb-seq has been a main application of gene-targeting approaches^3^. ActiveSVM could therefore provide a method for identifying minimal gene sets that can be applied to increase the scale of perturb-seq data collection.

### Defining brain region markers with spatial transcriptomics

Finally, to further demonstrate the generality of the ActiveSVM approach, we applied the procedure to identify minimal gene sets for classification of cells by spatial location in spatial transcriptomics data. Spatial transcriptomics is an emerging method for measuring mRNA expression within single cells while retaining spatial information and cellular proximity within a tissue. As an example, in SeqFish+, an imaging-based spatial transcriptomics method, cells are imaged in their tissue environment and mRNA transcripts are counted using single-molecule imaging of mRNA spots^18^. In all spatial transcriptomics applications, a common goal is the identification of genes that mark specific spatial locations within a tissue sample. Furthermore, spatial imaging methods are commonly limited by imaging time. Although Seqfish+ can profile 10,000 mRNA molecules per cell, the identification of reduced gene sets would reduce imaging time and throughput.

We applied ActiveSVM to identify genes associated with specific spatial locations in the mouse brain. We used a seqFISH+ dataset in which the authors profile 10,000 mRNA molecules in seven fields of view (FOV) in the mouse brain^18^. Fields of view correspond with spatially distinct regions of the mouse cortex as well as the subventricular zone and choroid plexus. We used the spatial location labels provided by ref. ^18^ to identify seven different brain locations (FOVs 1–5 correspond with cortex layers 2/3 through layer 6; FOV 6 with subventricular zone, and FOV 7 with choroid plexus). Applying the spatial location labels as class labels, we applied ActiveSVM to identify genes that could allow classification of single cells by their location in one of the seven classes and to define marker genes that correspond to specific spatial locations.

**Extended Data Fig. 1:**
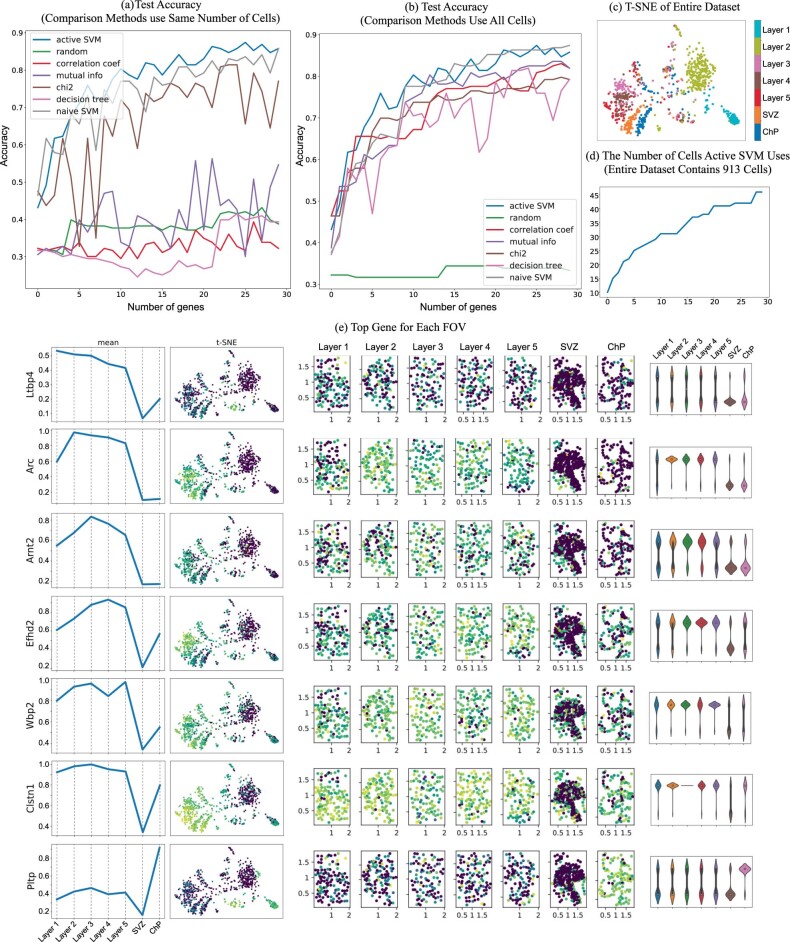
Application of ActiveSVM to identify region specific marker genes in the mouse brain with spatial transcriptomic data. The results of classification where cells are labeled according to fields of view (FOV). (a-b) test accuracy with min-complexity strategy, where comparison methods use the same number of cells as ActiveSVM in (a) and use the entire dataset in (b). Fields of view 1-5 correspond to 5 regions of the mouse cortex, additional fields of view are labeled SVZ (subventricular zone) and ChP (choroid plexus). (c) t SNE of cell transcriptomes for all cells (d) number of cells used per iteration (e) Sample of identified genes where each sub-panel shows mean expression across FOV/brain regions for selected gene, a tSNE plot colored by expression of selected gene, a violin plot of single cell gene expression values for selected gene in FOV/brain region, and spatial plots of each field of view where dots represents cells in 2D imaging slice, cells are colored by intensity of selected gene and units are in millimeters. Source data

We identified gene sets of <30 genes that enabled location classification with greater than 85% accuracy with min-complexity strategy (Extended Data Fig. 1a,b). ActiveSVM used only ten cells at each iteration but worked better than comparison methods who performed on the entire dataset (Extended Data Fig. 1b–d). In the spatial application, the result means that the ~30 genes are sufficient to classify single cells as belonging to one of the seven spatial classes. In Extended Data Fig. 1e, we show the mean expression of identified genes across cortical fields of view corresponding to a sweep through cortical layers 2/3 through 6 as well as the subventricular zone and the choroid plexus. Our analysis identifies markers Prex1 that are specific to the upper cortical layers of the brain. Efhd2, a calcium-binding protein linked to Alzheimer’s disease and dementia, was similarly expressed in lower cortical layers^43^. Finally, Pltp, a phospholipid transfer protein, was localized to the choroid plexus. In Extended Data Fig. 1e, we show the spatial distribution of these genes including their mean expression across regions, violin plots documenting expression distribution, and renderings of the single cells within the field of view and the relative expression of each gene. The spatial analysis demonstrates that a broad range of different experimental variables can be applied as labels. In each case ActiveSVM discovers genes that allow classification of cells according to labels and identifies interesting genes. Regional gene marker identification is a major task in seqFish data analysis and ActiveSVM is able to identify genes enriched in different brain regions automatically. Such spatial information could provide interesting insights into disease processes mediated by genes like Efhd2.

## Discussion

In this paper we introduce ActiveSVM as a feature selection procedure for discovering minimal gene sets in large single-cell mRNA-seq datasets. ActiveSVM extracts minimal gene sets through an iterative cell-state classification strategy. Conceptually, we refer to our strategy as active as it actively explores a dataset, identifying maximally informative cells for analysis. ActiveSVM specifically selects cells that fall within the margin of the SVM classifier and uses these poorly classified cells to search for maximally informative genes (features). In the machine learning literature, an algorithm is conventionally called active when it can directly query an oracle for data examples that meet a criteria^12,44^. In the tradition of active learning, our ActiveSVM procedure queries the SVM classifier for cells that have been misclassified and then expends computational resources to analyze all genes within that limited subset of cells to discover informative genes; thus, although our algorithm cannot query the biological system directly for cells that meet a specific criteria, the algorithm queries the dataset itself for informative examples.

Biologically, a recent work highlights the presence of a low-dimensional structure within the transcriptome^1^; the structure emerges in gene expression data as cells modulate their physiological state through gene expression programs or modules that contain large groups of genes. As genes within transcriptional modules have highly correlated expression, measurements performed on a small number of highly informative signature genes can be sufficient to infer the state of a cell^45^. Low-dimensional structures can be exploited to decrease measurement and analysis costs, as a small fraction of the transcriptome must be measured to infer the cellular state. We developed ActiveSVM as a scalable strategy for extracting high-information content genes within a sharply defined task: cell-state classification.

The ActiveSVM approach has several limitations in the current implementation. First we have developed ActiveSVM using a single classification method—the support vector machine—as the computational engine. Active learning methods can be applied more broadly to additional classification strategies such as neural network-based classification, as well as other types of analysis such as data clustering and gene regulatory network inference. Second, the method currently applies a supervised learning task—cell-state classification—to construct a minimal gene set. In datasets without explicit cell-state labels, we derive labels from unsupervised clustering of data. The active sampling strategy could be extended to a wider range of applications including fully unsupervised analysis methods and differentiation trajectory analysis. Third, in the current implementation, ActiveSVM selects single genes at each round. In some cases, highly informative gene pairs or triples might exist that can only be discovered through explicit combinatorial strategies that search for combinations of genes that increase classification accuracy at each iteration.

Although ActiveSVM currently focuses on the reduction of computational costs, we hope in the future to apply active sampling strategies directly at the point of measurement. In genomics, measurement resources often limit the scale of data acquisition. Single-cell mRNA-seq measurements are currently limited by sequencing and reagents costs. Similarly, spatial genomics methodologies are limited by imaging time. In future work we aim to develop strategies that can improve the on-line acquisition of single-cell data through active sampling. Active strategies could be implemented at the point of measurement by only sequencing or imaging the content of cells that meet a criteria, for example, cells identified as within a tumor microenvironment. Even more broadly, it might be possible to increase the information content of measurements by actually inducing biological systems to generate highly informative examples through designed experimental perturbations.

## Methods

### Identification of maximally informative cells

We formalize the ActiveSVM procedure and define mathematical rules that encode our specific gene and cell-selection strategies. In single-cell gene expression data we use ${x}_{i}^{(j)}\in {\mathbb{R}}$ to denote the measurement of the *j*th gene of the *i*th cell. We assume that the classification labels are given and consider that dataset ${\{{x}_{i},{y}_{i}\}}_{i\in \{1,\ldots,N\}}$ contains *N* cells with total *M* genes, where ${x}_{i}={[{x}_{i}^{(j)}]}_{j\in \{1,\ldots,M\}}$ and ${y}_{i}\in {\mathbb{Z}}$ are labels. The labels could be binary or multiclass, and can be derived from clustering. We also denote the gene expression vector of *i*th cell with part of genes as ${x}_{i}^{(D)}={[{x}_{i}^{(j)}]}_{j\in D}$, where *D* ⊂ {1, …, *M*}; we use *J* and *I* to refer to the set of selected genes and cell set.

We adopt the SVM classifier notation of one observation is ${h}_{w,b}({x}_{i}^{(D)})=g({w}^{T}{x}_{i}^{(D)}+b)$ for any *i* ∈ {1, 2, …, *N*} and *D* ⊂ {1, 2, …, *M*} with respect to observation $x\in {{\mathbb{R}}}^{| D| }$, where $w\in {{\mathbb{R}}}^{| D| }$ and $b\in {\mathbb{R}}$ are the margin and bias, respectively. Here, *g*(*z*) = 1 if *z* ≥ 0, otherwise *g*(*z*) = −1; the loss function is the Hinge loss^46^, ${{{{\rm{loss}}}}}_{i}=\max \{0,1-{y}_{i}({w}^{T}{x}_{i}^{(D)}+b)\}$, where ${y}_{i}\in {\mathbb{R}}$ is the ground-truth label of observation *x*_*i*_.

For the cell-selection strategy, we identify cells with the largest SVM classification loss. In SVM classification, samples separable in *n*-D are also separable in (*n* + 1)-D, as they are at least separated by the same boundary with zero at the (*n* + 1)th dimension; thus, to improve the classification accuracy with a new gene, we should only consider the misclassified cells. We identify such cells through analysis of the dual form of the classical SVM classification problem. After solving the primal optimization problem of soft margin SVM, we have the dual optimization problem with a non-negative Lagrange multiplier ${\alpha }_{i}\in {\mathbb{R}}$ for each inequality constraint^47^.

1 $$\begin{array}{ll}\mathop{\max }\limits_{\alpha }\quad &\mathop{\sum }\limits_{i=1}^{N}{\alpha }_{i}-\frac{1}{2}\mathop{\sum }\limits_{{i}_{1},{i}_{2}=1}^{N}{y}_{{i}_{1}}{y}_{{i}_{2}}{\alpha }_{{i}_{1}}{\alpha }_{{i}_{2}} < {x}_{{i}_{1}}^{(J)},{x}_{{i}_{2}}^{(J)} > \\ {{{\rm{s.t.}}}}\quad &0\le {\alpha }_{i}\le C\\ &\mathop{\sum }\limits_{i=1}^{N}{\alpha }_{i}{y}_{i}=0\end{array}$$

Here ${x}_{i}^{(J)}$ refers to the measurement of the *i*th cell with all selected genes, and $C\in {\mathbb{R}}$ is a hyperparameter we set to control the trade-offs between size of margin and margin violations when samples are non-separable.

We solve the optimal solution α^*^ and apply the Karush–Kuhn–Tucker dual-complementarity conditions^48^ to obtain the following results where $w\in {{\mathbb{R}}}^{| J| }$ and the intercept term $b\in {\mathbb{R}}$ are optimal.

2 $$\begin{array}{lll}{\alpha }_{i}^{* }=0&\Rightarrow &{y}_{i}({w}^{T}{x}_{i}^{(J)}+b) > 1\\ {\alpha }_{i}^{* }=C&\Rightarrow &{y}_{i}({w}^{T}{x}_{i}^{(J)}+b) < 1\\ 0 < {\alpha }_{i}^{* } < C&\Rightarrow &{y}_{i}({w}^{T}{x}_{i}^{(J)}+b)=1.\end{array}$$

Therefore, for each cell, the Lagrange multiplier *α*_*i*_ indicates whether the cell falls within the SVM margin defined by the vector *w*; *α*_*i*_ > 0 means *y*_*i*_(*w*^*T*^*x*_*i*_ + *b*) ≤ 1, that is, cells are on or inside the SVM margin. Hence we can directly select cells with *α*_*i*_ > 0. In practice, we typically only select cells with *α*_*i*_ = *C*, which indicates incorrectly classified cells.

Using this mathematical formulation, we develop two different versions of the ActiveSVM procedure, the min-complexity strategy and min-cell strategy, for distinct goals. The min-complexity strategy minimizes the time and memory consumption when computational resources are restricted or where a user desires to reduce run time. In the min-complexity strategy, a fixed number of cells is sampled among all misclassified cells and used as the cell set for gene selection in each iteration. A small number of cells can therefore be analyzed at each round and typically only few cells might be selected repeatedly. The two strategies are discussed in more detail below. We also developed random and balanced strategies for sampling cells across a series of cell-states with varying cell membership.

### Gene selection by maximizing margin rotation

To select maximally informative genes at each round, we analyze misclassified cells and identify genes that would induce the largest rotation of the classification margin. Our procedure is inspired by the active learning method, Expected Model Change^12^. We quantify rotation of the margin by calculating the twist angle induced in *w* when we add a new dimension (gene) to the classifier. Assume *J* is the set of genes we have selected so far. Once we add a gene into the ∣*J*∣-dimensional data space, the parameter *w* will have one more dimension. The rotation of margin measures how much *w* twists after adding the new dimension compared with the weight in the previous iteration.

Specifically, assume *J* is the set of genes we have selected so far. We derive the corresponding *w* from the optimal solution α^*^ (ref. ^47^). After solving the dual optimization problem (1), we have:

3 $$w=\mathop{\sum}\limits_{i\in I}{\alpha }_{i}^{* }{y}_{i}{x}_{i}^{(J)}.$$

We then pad *w* with zero to get a ∣*J* + 1∣-dimensional weight *w*_padded_, whose first ∣*J*∣ dimensions is *w* and the ∣*J* + 1∣th dimension is zero.

For each candidate gene *j*, we train a new ∣*J* + 1∣-dimensional SVM model and have weight *w*_*j*_, where *j* ∈ {1, …, *M*}⧹*J*. That is to say, for candidate gene *j*, we solve the dual optimization problem (4) and find a new optimal multiplier α^*(*j*)^. Note that we only use the selected cells here, *i*_1_, *i*_2_ ∈ *I*.

4 $$\begin{array}{ll}\mathop{\max }\limits_{\alpha }\quad &\mathop{\sum}\limits_{i\in I}{\alpha }_{i}^{(j)}-\frac{1}{2}\mathop{\sum}\limits_{{i}_{1},{i}_{2}\in I}{y}_{{i}_{1}}{y}_{{i}_{2}}{\alpha }_{{i}_{1}}^{(j)}{\alpha }_{{i}_{2}}^{(j)}\langle {x}_{{i}_{1}}^{(J\cup \{j\})},{x}_{{i}_{2}}^{(J\cup \{j\})}\rangle \\ {{{\rm{s.t.}}}}\quad &0\le {\alpha }_{i}^{(j)}\le C\\ &\mathop{\sum}\limits_{i\in I}{\alpha }_{i}^{(j)}{y}_{i}=0\\ \end{array}$$

Then we have *w*_*j*_ as shown in equation (5):

5 $${w}_{j}=\mathop{\sum}\limits_{i\in I}{\alpha }_{i}^{* (j)}{y}_{i}{x}_{i}^{(J\cup \{j\})}$$

The angle *θ*_*j*_ between *w*_*j*_ and *w*_padded_ is the expected angle the margin rotates, corresponding to the *j*th candidate gene. Then the *j*th gene with largest angle *θ*_*j*_ will be selected. We measure the angle between two vectors using cosine similarity^49^:

6 $${\vartheta }_{j}=\arccos \cos {\vartheta }_{j}=\arccos \frac{\langle {w}_{j},{w}_{padded}\rangle }{\parallel {w}_{j}\parallel \parallel {w}_{padded}\parallel }$$

Thus, a new gene, which maximizes *ϑ*_*j*_, is selected to maximize the expected model change.

### Multiclass ActiveSVM

For multiclass classification, the SVM is handled according to a one-versus-the-rest scheme, where a separate classifier is fit for each class, against all other classes. Margin rotation is represented as the sum of weight components in each class dimension. Hence with *Z* classes, we get *Z* weight components corresponding to *Z* one-versus-the-rest classification decision boundaries. Assume the weight component for class *z* of the previous ∣*J*∣-dimensional SVM model is *w*^(*z*)^. Denote the ∣*J* + 1∣-dimensional weight after zero-padding of *w*^(*z*)^ as ${w}_{\mathrm{padded}}^{(z)}$ and the new ∣*J* + 1∣-dimensional weight component of class *z* with *j*th gene as ${w}_{j}^{(z)}$, where *z* ∈ 1, …, *Z*. We then have:

7 $${\vartheta }_{j}^{(z)}=\arccos \cos {\vartheta }_{j}^{(z)}=\arccos \frac{\langle {w}_{j}^{(z)},{w}_{padded}^{(z)}\rangle }{\parallel {w}_{j}^{(z)}\parallel \parallel {w}_{padded}^{(z)}\parallel }$$

8 $${\vartheta }_{j}=\mathop{\sum }\limits_{z=1}^{Z}{\vartheta }_{j}^{(z)}$$

### Min-cell and min-complexity cell selection strategies

In the min-cell strategy, to reduce the number of unique cells required, the misclassified cells already used in previous steps are given the highest priority to be reselected. The min-cell strategy therefore attempts to reuse cells across rounds of iteration and aims to minimize the total number of unique cells we acquire during the entire procedure. The min-cell strategy can be applied to limit the number of cells required to perform the analysis in settings where cell acquisition might be limiting, including in the analysis of rare cell populations or in clinical datasets.

For the min-cell strategy, assume we select *c* cells for each iteration and there are *a* + *b* misclassified cells at the current iteration, where *a* cells have been used at least once in previous iterations, whereas *b* cells are new cells. If *a* ≥ *c*, we do not need to add any new cells to current cell set. If *a* < *c*, we sample *c* − *a* cells among the *b* new cells. The algorithm then uses the whole selected cell set for the next gene selection step. When using the min-cell strategy, cells tend to be reused many times and the curve of number of unique cells we acquire converges to a fixed value along with the number of genes we select. In experiments, the number of cells selected for each step, *c*, is a hyperparameter set by the user. Typically, the parameter can be set to a small number using the min-complexity strategy, as a sufficient number of new cells is considered in the procedure. Selecting a small number of cells each round reduces computational complexity. In the min-cell strategy it can be advantageous to select a larger number of total cells to guarantee diversity of training cells while still bounding the total number of cells used.

### Balancing cell sampling across cell classes

In addition to the min-cell and min-complexity options, we also include two version of cell sampling strategies. The first one is uniform, random sampling. Another option is class-balanced sampling, which can be applied to balance sampling across a series of cell classes. In the balanced strategy we sample a fixed number of cells from each cell class, whereas for classes with insufficient cells we sample all of the cells in the class. Mathematically, assume there are *Z* classes and *S* is the set of all misclassified cells this step. We should sample $c^{\prime}$ cells from a candidate cell set $S^{\prime}$ for the current iteration. In min-complexity strategy, $c^{\prime} =c$ and the candidate cell set $S^{\prime}$ should be *S* itself. For the min-cell strategy, $c^{\prime} =c-\min \{c,| I\cap S| \}$, where *I* is the cell set before current iteration, and the candidate cell set $S^{\prime} =S\setminus I$. Assume $S^{\prime} ={\cup }_{z = 1}^{Z}{S}_{z}^{\prime}$, where ${S}_{z}^{\prime}$ are the set of cells in class *z* and $| {S}_{z}^{\prime}| \le | {S}_{(z+1)}^{\prime}|$ for any *z* ∈ {1, 2, ... , *Z* − 1}. We sample cells in order from class 1 to class *Z* and denote *P*_*z*_ as the union set of all selected cells from all classes after class *z*. Then for class *z*, if $| {S}_{z}^{\prime}| \le (| S^{\prime} | -| {P}_{z-1}| )/(Z-z+1)$, we select all cells in ${S}_{z}^{\prime}$. Otherwise, if $| {S}_{z}^{\prime}| > (| S^{\prime} | -| {P}_{z-1}| )/(Z-z+1)$, we randomly sample $(| S^{\prime} | -| {P}_{z-1}| )/(Z-z+1)$ cells in ${S}_{z}^{\prime}$. The procedure repeats for all classes and then we have *P*_*Z*_ as the cells we select at this iteration.

### Incorporation of cell labels derived from unsupervised analysis, experimental conditions or biological knowledge

The goal of ActiveSVM is to discover minimal gene sets for extracting biological information from single-cell datasets. To define minimal gene sets, we apply a classification task in which we find genes that enable a SVM classifier to distinguish single cells with different labels (*y*_*i*_). In practice, explicit cell-type labels are often not known for a dataset. An extremely common work-flow in single-cell genomics applies Louvain clustering algorithms to identify cell classes and visualizes these cell classes in UMAP or t-SNE plots^50,51^. The cell clusters that are output by clustering work-flows in commonly used single-cell analysis frameworks provide a natural set of labels for downstream analysis. In fact, ActiveSVM can then identify specific marker genes for interpreting the identified cell-clusters and determining their biological identify. More broadly, cell-class labels can be quite general including the identity of a genetic perturbation (Fig. 6), the spatial location of a cell (Extended Data Fig. 1). We can imagine the application of ActiveSVM to a broad set of additional labels including membership to a differentiation trajectory or lineage tree^52^.

### Memory complexity of ActiveSVM

One of the key contribution of ActiveSVM is that it substantially saves memory usage because only a small part of data is used at each iteration. The entire dataset can be stored in disk and the algorithm only loads two small matrices into memory, a *N* × ∣*J*∣ matrix of all cells with the currently selected genes and a ∣*I*∣ × *M* matrix of the cell set with all genes. The memory complexity is ${{{\mathcal{O}}}}(M+N)$, whereas the memory complexity of algorithms using the entire dataset is at least ${{{\mathcal{O}}}}(MN)$. The min-cell strategy minimizes the total number of unique cells acquired to reduce the cost of data measurement, acquisition and storage.

### Time complexity of ActiveSVM

The time complexity of the complete procedure depends primarily on the training of SVM. The standard time complexity of SVM training is usually ${{{\mathcal{O}}}}(M{N}^{2})$^53^. Assume that we plan to select $k\in {\mathbb{N}}$ genes in total and use the cell set *I*_*i*_ of poorly classified cells at *i*th iteration, where *k*, *k*^2^ ≪ *M* and ∣*I*_*i*_∣, ∣*I*_*i*_∣^2^ ≪ *N* are constants the computational complexity of ActiveSVM is then:

$${{{\mathcal{O}}}}(\mathop{\sum }\limits_{i=1}^{k}(i\times {N}^{2}+(M-i)\times (i+1)\times | {I}_{i}{| }^{2})) \approx {{{\mathcal{O}}}}({N}^{2}+M).$$

The key reduction in total complexity occurs because each step is performed using *N* cells with of order *k*, *k*^2^ ≪ *M* genes or using order *M* genes with ∣*I*_*i*_∣ cells. The polynomial ${{{\mathcal{O}}}}(M{N}^{2})$ is therefore reduced to two separate steps: ${{{\mathcal{O}}}}({N}^{2})$ and ${{{\mathcal{O}}}}(M)$.

In practice, we implement ActiveSVM using the linear SVM library LIBLINEAR^54^, whose time complexity is ${{{\mathcal{O}}}}(MN)$; the corresponding time complexity of ActiveSVM with LIBLINEAR is:

$${{{\mathcal{O}}}}(\mathop{\sum }\limits_{i=1}^{k}(i\times N+(M-i)\times (i+1)\times | {I}_{i}| )) \approx {{{\mathcal{O}}}}(N+M).$$

In the gene selection part, the margin rotation angles of all candidate genes can be computed in parallel, which also accelerates the algorithm. The complexity provides a substantial improvement in marker gene selection methods especially for large-scale datasets.

### Computational Infrastructure

To analyze computational requirements of ActiveSVM, we performed analysis using an r5n.24xlarge, a type of EC2 virtual server instance on AWS, with 96 virtual central processing units and 768 GiB memory on Linux system. The instance allowed us to track run time and memory usage. As an example, for the largest dataset analysis, we applied ActiveSVM to select 50 genes on the largest dataset, mouse brain megacell dataset, which contains 1,306,127 cells and 27,998 genes, using ActiveSVM and some other popular feature selected methods, including correlation coefficient, mutual information, feature importance by decision tree and conventional SVM. The peak memory usage of ActiveSVM is 2,111 MB, whereas other methods all consume more than 78,600 MB. The run time of the min-complexity method is about 69 min, whereas that of the min-cell method is about 243 min. Each comparison method takes more than four days on the same server machine. The run time and peak memory usage of ActiveSVM on all six datasets are shown in Supplementary Table 1. The ActiveSVM package used for the brain megacell dataset loads only the selected genes and cells, into computer memory at each iteration, while the code for the other two experiments loads the entire dataset when calling the package. Pipelines demonstrating both settings are provided in the 'Code availability' section.

### Data preprocessing

#### PBMC, Tabula Muris and multiple myeloma

The PBMC, Tabula Muris and multiple myeloma datasets were preprocessed for ref. ^16^ via column normalizaition. In each experiment, we removed the columns and rows where all values are zero. Subsequently, gene expression matrices were first-columns normalized and log transformed. For a cell *i*, each gene ${x}_{i}^{(j)}$ (gene *j* in cell *i*) is first normalized as ${\tilde{x}}_{i}^{(j)}=\frac{{x}_{i}^{(j)}}{\mathop{\sum }\nolimits_{i = 1}^{M}{x}_{i}^{(j)}}$ where *M* is the number of genes in the transcriptome; we then performed *l*^2^-normalization for each cell, which means scaling each cell vector individually to unit *l*^2^-norm.

#### Mega-cell dataset, perturb-seq and spatial transcritomics

We removed the columns and rows where all values are zero for Mega-cell, perturb-seq and spatial transcritomics. We then performed *l*^2^-normalization along each cell.

### Calculation of Confidence Intervals

Confidence intervals were estimated using a proportion confidence interval^55^ as ${{{\rm{interval}}}}=z\sqrt{\frac{\epsilon * (1-\epsilon )}{N}\left.\right)}$, where *z* = 1.96 for 95% confidence and *N* is the number of cells and *ϵ* the observed error.

### Supplementary information

Supplementary Information The algorithm of ActiveSVM, Supplementary Figs. 1 and 2 show the experimental results of the min-cell strategy on the Tabula Muris and multiple myeloma datasets. Supplementary Table 1 covers run time and memory consumptions, as well as further details on the experiments, including data preprocessing and confidence intervals. Supplementary Tables 2–4 cover the parameters of each experiment.

### Source data

Source Data Fig. 2 There are six folders for six subfigures. Fig. 2a: the min-cell strategy accuracy versus the number of genes selected by ActiveSVM and all comparison methods. Fig. 2b: the min-complexity strategy accuracy versus the number of genes selected by ActiveSVM and all comparison methods. Fig. 2c: the t-SNE coordinates and labels. Fig. 2d: the number of cells acquired by min-cell strategy versus the number of genes selected. Fig. 2e: t-SNE coordinates and the partial processed data matrix used to plot top gene markers. Fig. 2f: t-SNE coordinates and the partial processed data matrix for additional gene markers.

Source Data Fig. 3 There are seven folders for seven subfigures. Fig. 3a: the min-complexity strategy accuracy versus the number of genes selected by ActiveSVM and all comparison methods. Fig. 3b: the min-cell strategy accuracy versus the number of genes selected by ActiveSVM and all comparison methods. Fig. 3c: the number of cells acquired by min-cell strategy versus the number of genes selected. Fig. 3d: the t-SNE coordinates and labels. Fig. 3e: t-SNE coordinates and the partial processed data matrix used to plot some comparison gene markers from original paper. Fig. 3f: t-SNE coordinates and the partial processed data matrix for top gene markers we selected. Fig. 3g: the coefficient matrix between the gene markers we selected and comparison markers from the original paper.

Source Data Fig. 4 There are five folders for five subfigures. Fig. 4a: the min-complexity strategy accuracy versus the number of genes selected by ActiveSVM and all comparison methods. Fig. 4b: the t-SNE coordinates and labels. Fig. 4c: the t-SNE coordinates projected from the matrix with only genes selected by random sample and labels. Fig. 4d: the t-SNE coordinates projected from matrix with only genes selected by balance sample and labels. Fig. 4e: the t-SNE coordinates and the partial processed data matrix used to plot top gene markers.

Source Data Fig. 5 There are five folders for five subfigures. Fig. 5a: the min-complexity strategy accuracy versus the number of genes selected by ActiveSVM and all comparison methods. Fig. 5b: the t-SNE coordinates and labels. Fig. 5c: the t-SNE coordinates projected from the matrix with only genes selected by random sample and labels. Fig. 5d: the t-SNE coordinates projected from matrix with only genes selected by balance sample and labels. Fig. 5e: the t-SNE coordinates and the partial processed data matrix used to plot top gene markers.

Source Data Fig. 6 There are four folders for four subfigures. ED3a: the accuracy versus the number of genes selected by ActiveSVM and all comparison methods, compared with the same number of cells used. ED3b: the accuracy versus the number of genes selected by ActiveSVM and all comparison methods, where comparison methods use the entire dataset. ED3c: the coefficient matrix between gene markers we selected. ED3d: the processed partial data matrix used for distribution plots of top gene markers we selected.

Source Data Extended Data Fig. 1 There are five folders for five subfigures. ED4a: the accuracy versus the number of genes selected by ActiveSVM and all comparison methods, compared with the same number of cells used. ED4b: the accuracy versus the number of genes selected by ActiveSVM and all comparison methods, where comparison methods use the entire dataset. ED4c: the t-SNE coordinates and labels. ED4d: number of acquired cells versus number of genes selected. Extended Data Fig. 4e: the t-SNE coordinates, the processed partial data matrix used for distribution plots of top gene markers we selected, and the FOV coordinates.

## Data Availability

All of the data used in the paper have been previously published. The PBMC Single-cell RNA-seq data have been deposited in the Short Read Archive under accession no. SRP073767 by the authors of ref. ^13^. Data are also available at http://support.10xgenomics.com/single-cell/datasets. The original Tabula Muris dataset is available at https://figshare.com/projects/Tabula_Muris_Transcriptomic_characterization_of_20_organs_and_tissues_from_Mus_musculus_at_single_cell_resolution/27733. The original multiple myeloma PBMC data, which contain two healthy donors and four multiple myeloma donors, are available at https://figshare.com/articles/dataset/PopAlign_Data/11837097/3. The 10x Genomics Megacell dataset is available at http://support.10xgenomics.com/single-cell/datasets. The perturb-seq dataset^17^ is available at https://www.ncbi.nlm.nih.gov/geo/query/acc.cgi?acc=GSM2396856 The spatial transcriptomics data^18^ are available at https://github.com/CaiGroup/seqFISH-PLUS. Source Data are provided with this paper.

## Code availability

Our method is integrated as an installable Python package called ActiveSVC. The installation instructions and user guidance are shown at https://pypi.org/project/activeSVC. The source codes of activeSVC and some demo examples are publicly available on GitHub at https://github.com/xqchen/activeSVC and Zenodo^56^. We also created a Google colaboratory project demonstrating three examples: the PBMC demo is at https://colab.research.google.com/drive/16h8hsnJ3ukTWAPnCB581dwj-nN5oopyM?usp=sharing, the Tabula Muris demo is at https://colab.research.google.com/drive/1SLehIKIQqpjK6BzEKc9m0y3uJ_LBqRzA?usp=sharing, and the PBMC cross-validation^57^ demo is at https://colab.research.google.com/drive/1fhQ8GD3NyzB3w0vof9WimXK6BLqDNuDC?usp=sharing.
