# Supplementary material for: Minimal gene set discovery in single-cell mRNA-seq datasets with ActiveSVM
Source: Nat Comput Sci. 2022 Jun 27;2(6):387–98. doi: 10.1038/s43588-022-00263-8 (PMC10766518; doi:10.1038/s43588-022-00263-8)
Supplement: Supplementary file 1 — The algorithm of ActiveSVM, Supplementary Figs. 1 and 2 show the experimental results of the min-cell strategy on the Tabula Muris and multiple myeloma datasets. Supplementary Table 1 covers run time and memory consumptions, as well as further details on the experiments, including data preprocessing and confidence intervals. Supplementary Tables 2–4 cover the parameters of each experiment. [file 43588_2022_263_MOESM1_ESM.pdf]

nature portfolio

https://doi.org/10.1038/s43588-022-00263-8

Supplementary information

Minimal gene set discovery in single-cell mRNA-seq datasets with ActiveSVM

In the format provided by the authors and unedited

Supplementary Information

Algorithm

For notation, in single-cell gene expression data, we use $x_i^{(j)} \in \mathbb{R}$ to denote the measurement of the j-th gene of the i-th cell. We assume the classification labels are given and consider a data-set $\{x_i, y_i\}_{i\in\{1,\dots,N\}}$ contains N cells with total M genes, where $x_i = [x_i^{(j)}]_{j\in\{1,\dots,M\}}$ and $y_i \in \mathbb{Z}$ are labels. The labels could be binary or multi-class and can be derived from clustering. We also denote the gene expression vector of i-th cell with part of genes as $x_i^{(D)} = [x_i^{(j)}]_{j\in D}$, where $D \subset \{1,\dots,M\}$. And we use J and I to refer to the set of selected genes and cell set.

We adopt the SVM classifier notation of one observation is $h_{w,b}(x_i^{(D)}) = g(w^T x_i^{(D)} + b)$ for any $i \in \{1, 2, \dots, N\}$ and $D \subset \{1, 2, \dots, M\}$ with respect to observation $x \in \mathbb{R}^{|D|}$, where $w \in \mathbb{R}^{|D|}$ and $b \in \mathbb{R}$ are parameters (the margin and bias respectively). Here, $g(z) = 1$ if $z \geq 0$, and $g(z) = -1$ otherwise. And the loss function is Hinge Loss[1] $\text{loss}_i = \max\{0, 1 - y_i(w^T x_i^{(D)} + b)\}$, where $y_i \in \mathbb{R}$ is the ground truth label of observation x_i.

Algorithm 1: Active Linear SVM Gene Selection

Input: $c, k \in \mathbb{N}$, $J = \emptyset$
Output: J
Randomly or 'balanced' select c cells $I \subset \{1,\dots,N\}$, $|I| = c$
Train 1-D SVM models on training set I for each candidate gene: $\{h_{w,b}^{(j)}\}_{j\in\{1,\dots,M\}}$
$loss_j = \sum_{i\in I} \max\{0, 1 - y_i(w^T x_i^{(j)} + b)\}$
Select one gene $j_0 \in \{1,\dots,M\}$ with lowest $loss_j$
$J = J \cup \{j_0\}$
repeat
  Optimize (1) and get optimal solution $\{\alpha_i^*\}_{i=1}^N$
  Get the the set of misclassified cells $S \subset \{1,\dots,N\}$ with $\alpha_i^* = C$
  if *min-complexity* **then**
    Randomly or 'balanced' select c cells $I \subset S$, where $|I| = c$;
  else
    if *min-cell* **then**
      $c' = \min\{c, |I \cap S|\}$;
      Randomly or 'balanced' select $c - c'$ cells $I' \subset S \setminus I$, where $|I'| = c - c'$;
      $I = I \cup I'$
    end
  end
  $w = \sum_{i\subset I} \alpha_i^* y_i x_i^{(J)}$
  $w_{padded} = [w, 0]$
  For each $j \in \{1,\dots,M\} \setminus J$, optimize (4) and get optimal solution $\{\alpha_i^{*(j)}\}_{i\in I}$
  $w_j = \sum_{i\in I} \alpha_i^{*(j)} y_i x_i^{(J\cup\{j\})}$
  $\vartheta_j = \arccos\cos\vartheta_j = \arccos \frac{<w_j, w_{padded}>}{\|w_j\|\|w_{padded}\|}$
  Select one gene $j^* \in \{1,\dots,M\} \setminus J$ with largest ϑ_j
  $J = J \cup \{j^*\}$
until $|J| \geq k$

Supplementary Figures

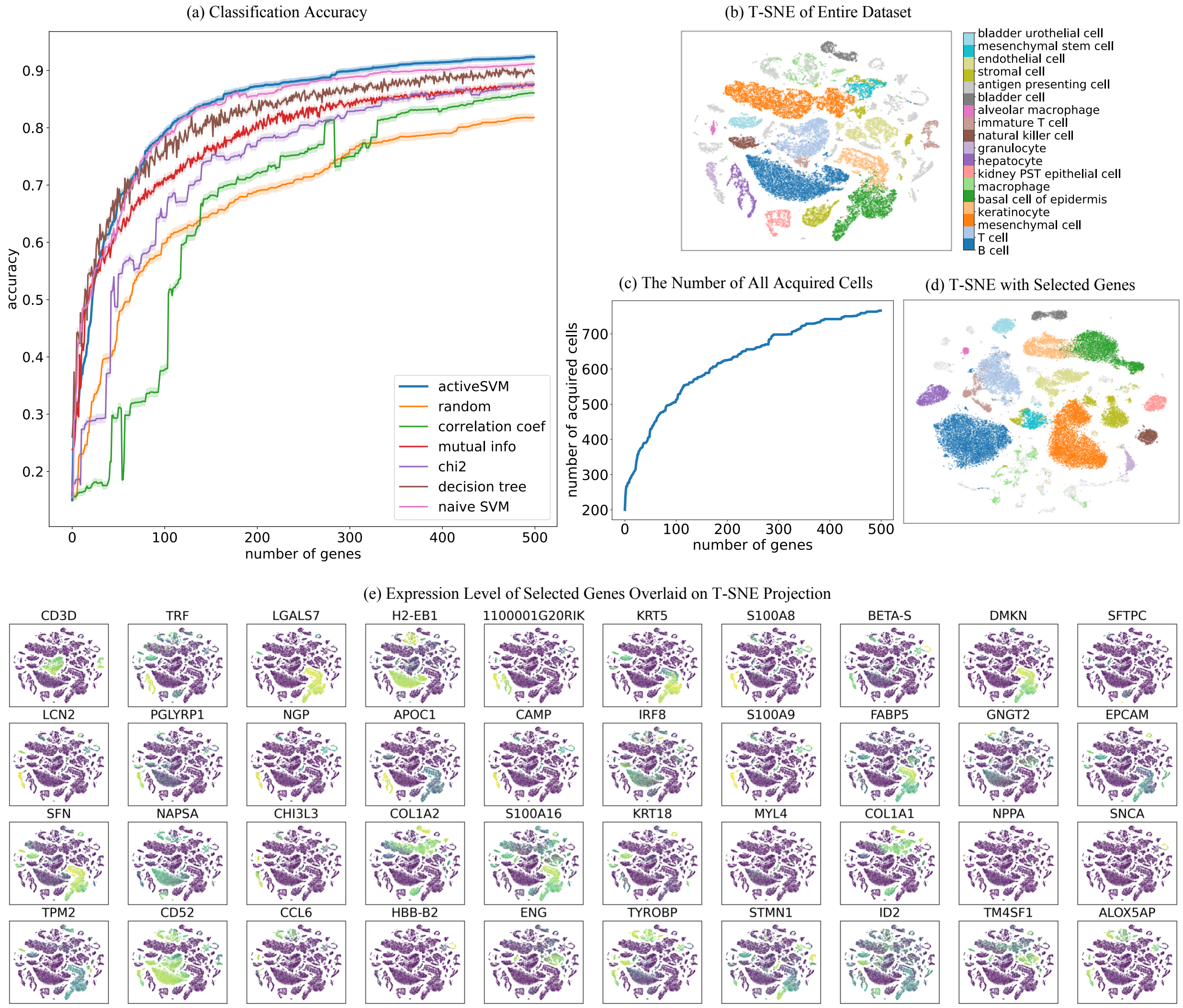

Supplementary Figure 1: Minimal gene sets for cell-type classification in the Tabula Muris mouse tissue survey. Classification results of 500 genes selected using the min-cell strategy with 200 cells per iteration. The subplots contain: (a) classification accuracy vs gene set size; (b) the t-SNE plots of the entire filtered dataset; (c) the total number of unique cells used vs gene set size; (d) the t-SNE plots of the gene set selected with 'balanced' sampling; (e) the expression level overlaid on t-SNE projection for genes selected.

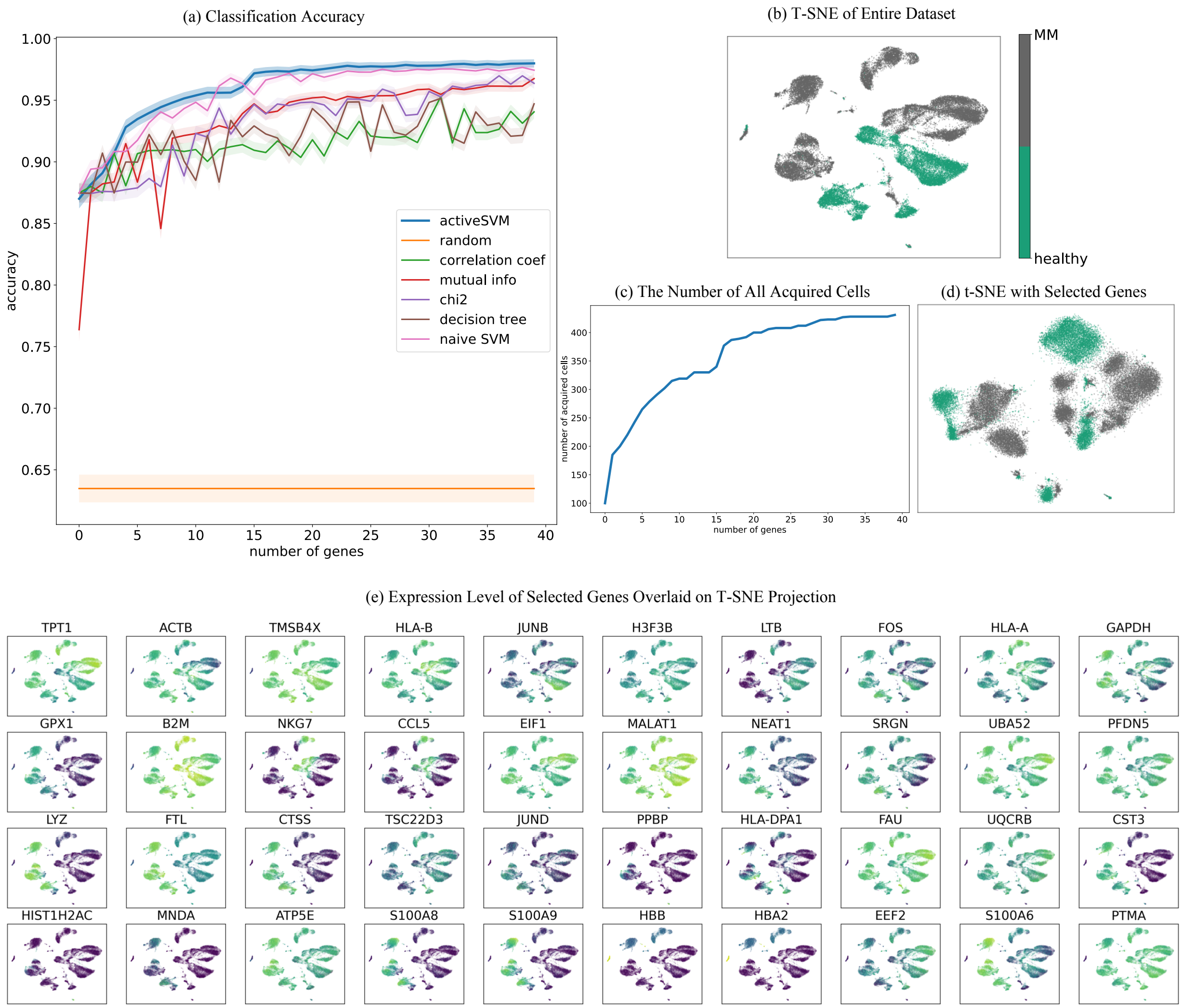

Supplementary Figure 2: Gene set selection for healthy vs disease classification in multiple myeloma dataset. Classification results of 40 genes selected using min-cell strategy with 100 cells per iteration. (a) classification accuracy vs gene set size; (b) the t-SNE plots of the entire filtered dataset; (c) the total number of unique cells used vs gene set size; (d) the t-SNE plots of the gene set selected with 'balanced' sampling; (e) the expression level overlaid on t-SNE projection for genes selected.

Computational Time Consumption

To analyze computational requirements of ActiveSVM, we performed analysis using an r5n.24xlarge, a type of EC2 virtual server instance on AWS, with 96 virtual central processing units (vCPU) and 768 GiB memory on Linux system. The run time and peak memory usage of ActiveSVM on all six datasets are shown in Supplementary Table 1.

Supplementary Table 1: Run Time and Peak Memory Usage of ActiveSVM.

	matrix size (cells, genes)	min-complexity run time (s/gene)	min-cell run time (s/gene)	memory (MB)	unique cells (min-cell)
mouse megacell	(1306127, 27998)	4142/50	14580/50	2111	712
PBMC	(10194, 6915)	121/50	176/20	1325	298
Tabula Muris	(55656, 8661)	737/150	7701/100	1093	779
MM	(35159, 32527)	127/40	449/40	1616	445
seqFish	(913, 10000)	33/30	728/30	887	428
perturb-seq	(10895, 15976)	3424/50		9493	3827

Parameter Optimization

For conventional and ActiveSVM, we found the approximately optimal parameter by grid-search [2] across lists of candidate values for some key parameters in the framework of 3-fold cross validation [3]. The optimal parameters were fixed during all iterations. For the comparison methods, we use 3-fold cross validation grid-search to obtain the optimal parameters at each single iteration. We also implemented the algorithms called `min_complexity_cv` and `min_acquisition_cv` that apply grid-search and cross validation for each single SVM trained in each iteration (see Code Availability).

Here we provide the algorithm parameters we used for ActiveSVM in Supplementary Table 2-4. Besides the training set and test set, there are 15 user-defined hyper-parameters in ActiveSVM, five of which are about the feature selection procedure and the other ten are commonly-used parameters for linear SVM classifier. The detailed description about all parameters of ActiveSVM are detailed described in the integrated package page https://pypi.org/project/activeSVC/.

As for comparison methods, correlation coefficient, mutual information, and chi-squared methods don't have specific parameters to set. We implemented them with scikit-learn[4] package 'SelectKBest'. For feature importance scores from decision tree and naive SVM, we did grid-search on key parameters based on 3-fold cross validation at each step. The parameters of decision tree are $criterion$ and $min\_samples\_leaf$ and of naive SVM are tol and C.

Supplementary Table 2: Parameters of ActiveSVM (PBMC and mouse megacell datasets).

	PBMC (min-complexity)	PBMC (min-cell)	mouse megacell (min-complexity)	mouse megacell (min-cell)
$num\_features$	50	20	50	50
$num\_samples$	20	100	20	100
$init\_features$	1	1	1	1
$init\_samples$	20	200	20	100
$balance$	True/False	True	True	True
$penalty$	'l2'	'l2'	'l2'	'l2'
$loss$	squared_hinge	squared_hinge	squared_hinge	squared_hinge
$dual$	True	True	True	True
tol	1e-4	1e-4	1e-4	1e-4
C	1.0	1.0	1.0	1.0
$fit\_intercept$	True	True	True	True
$intercept\_scaling$	1	1	1	1
$class\_weight$	None	None	'balanced'	'balanced'
$random\_state$	None	None	None	None
max_iter	1000	1000	1000	1000

Supplementary Table 3: Parameters of ActiveSVM (Tabula Muris and MM datasets).

	Tabula Muris (min-complexity)	Tabula Muris (min-cell)	MM (min-complexity)	MM (min-cell)
$num\_features$	150	500	40	40
$num\_samples$	20	200	20	100
$init\_features$	1	1	1	1
$init\_samples$	20	200	20	100
$balance$	True/False	False	True/False	False
$penalty$	'l2'	'l2'	'l2'	'l2'
$loss$	squared_hinge	squared_hinge	squared_hinge	squared_hinge
$dual$	True	True	True	True
tol	1e-4	1e-4	1e-4	1e-4
C	1.0	1.0	1.0	1.0
$fit\_intercept$	True	True	True	True
$intercept\_scaling$	1	1	1	1
$class\_weight$	None	None	None	None
$random\_state$	None	None	None	None
max_iter	1000	1000	1000	1000

Supplementary Table 4: Parameters of ActiveSVM (perturb-seq and seqFish datasets).

	perturb-seq (min-complexity)	seqFish (min-complexity)
$num\_features$	50	30
$num\_samples$	500	10
$init\_features$	1	1
$init\_samples$	1000	10
$balance$	True	False
$penalty$	'l2'	'l2'
$loss$	squared_hinge	squared_hinge
$dual$	True	True
tol	1e-6	1
C	1.0	10
$fit\_intercept$	True	True
$intercept\_scaling$	1	1
$class\_weight$	'balanced'	None
$random\_state$	None	None
max_iter	1,000,000	100,000

References

[1] L. Rosasco, E. De Vito, A. Caponnetto, M. Piana, and A. Verri, "Are loss functions all the same?," *Neural computation*, vol. 16, no. 5, pp. 1063–1076, 2004.

[2] I. Syarif, A. Prugel-Bennett, and G. Wills, "Svm parameter optimization using grid search and genetic algorithm to improve classification performance," *Telkomnika*, vol. 14, no. 4, p. 1502, 2016.

[3] S. Arlot and A. Celisse, "A survey of cross-validation procedures for model selection," *Statistics surveys*, vol. 4, pp. 40–79, 2010.

[4] F. Pedregosa, G. Varoquaux, A. Gramfort, V. Michel, B. Thirion, O. Grisel, M. Blondel, P. Prettenhofer, R. Weiss, V. Dubourg, J. Vanderplas, A. Passos, D. Cournapeau, M. Brucher, M. Perrot, and E. Duchesnay, "Scikit-learn: Machine learning in Python," *Journal of Machine Learning Research*, vol. 12, pp. 2825–2830, 2011.
