# Supplementary figures and images for: Minimal gene set discovery in single-cell mRNA-seq datasets with ActiveSVM
Source: Nat Comput Sci. 2022 Jun 27;2(6):387–98. doi: 10.1038/s43588-022-00263-8 (PMC10766518; doi:10.1038/s43588-022-00263-8)

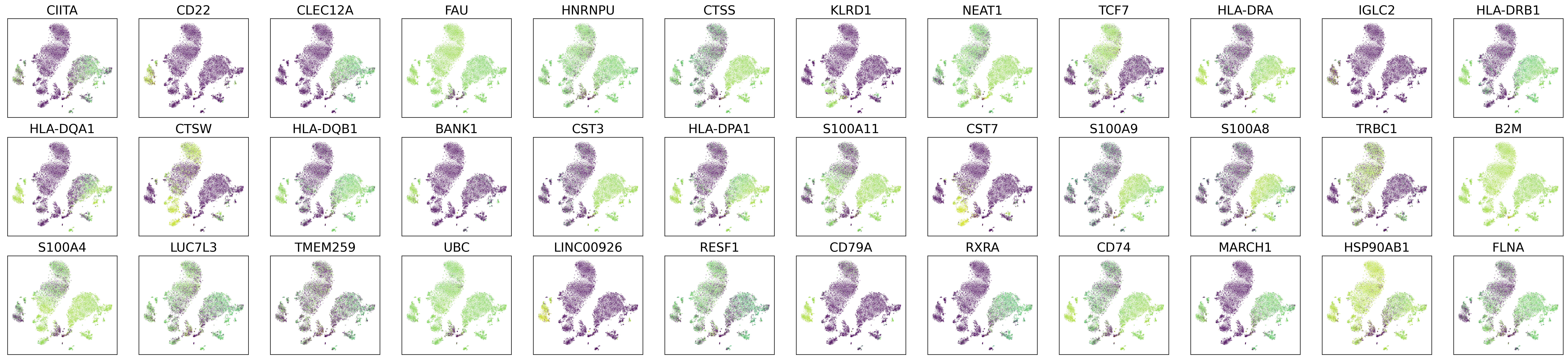

Supplement: Supplementary file 2 — There are six folders for six subfigures. Fig. 2a: the min-cell strategy accuracy versus the number of genes selected by ActiveSVM and all comparison methods. Fig. 2b: the min-complexity strategy accuracy versus the number of genes selected by ActiveSVM and all comparison methods. Fig. 2c: the t-SNE coordinates and labels. Fig. 2d: the number of cells acquired by min-cell strategy versus the number of genes selected. Fig. 2e: t-SNE coordinates and the partial processed data matrix used to plot top gene markers. Fig. 2f: t-SNE coordinates and the partial processed data matrix for additional gene markers. [file 43588_2022_263_MOESM2_ESM.zip › figure2/figure2f/gene 05.20.55.png]
